# Community Health Workers as Social Marketers of Injectable Contraceptives: A Case Study from Ethiopia

**DOI:** 10.9745/GHSP-D-16-00344

**Published:** 2017-03-08

**Authors:** Karen Weidert, Amanuel Gessessew, Suzanne Bell, Hagos Godefay, Ndola Prata

**Affiliations:** a University of California at Berkeley, School of Public Health, Bixby Center for Population Health and Sustainability, Berkeley, CA, USA.; b Mekelle University College of Health Sciences, Mekelle, Ethiopia.; c Johns Hopkins Bloomberg School of Public Health, Baltimore, MD, USA.; d Tigray Regional Health Bureau, Mekelle, Tigray, Ethiopia.

## Abstract

Ethiopia has made notable progress in increasing awareness and knowledge of family planning and is considered a success story among funders and program planners. Yet unmet need among rural women (28.6%) is almost double that of urban women (15.5%), with a wide gap in total fertility rate depending on urban (2.6) or rural (5.5) residence. This study investigates the impact of a service delivery model that combines community-based distribution (CBD) of contraception with social marketing in Tigray, Ethiopia, to create a more sustainable approach to CBD. Between September 2011 and October 2013, 626 volunteer CHWs were recruited and trained to administer depot medroxyprogesterone acetate (DMPA) injections and provide counseling and referrals to the health post for other methods; the project implementation period ended in June 2014. The CHWs received a supply of DMPA injections in the form of a microloan from a drug revolving fund; the CHWs charged women a minimal fee (5 birr, or US$0.29), determined based on willingness-to-pay data, for each DMPA injection; and the CHWs returned part of the fee (3 birr) to the drug revolving fund while keeping the remaining portion (2 birr). The CHWs also promoted demand for family planning through door-to-door outreach and community meetings. Existing health extension workers (HEWs) provided regular supervision of the CHWs, supplemented by in-depth supervision visits from study coordinators. Baseline and endline representative surveys of women of reproductive age, as well as of participating CHWs, were conducted. In addition, DMPA provision data from the CHWs were collected. Between October 2011 and June 2014, the CHWs served in total 8,604 women and administered an estimated 15,410 DMPA injections, equivalent to providing 3,853 couple-years of protection. There was a 25% significant increase in contraceptive use among surveyed women, from 30.1% at baseline to 37.7% at endline, with DMPA use largely responsible for this increase. Changes in quality of family planning markers from baseline suggested services improved between baseline and endline: nearly 50% more women reported being told about side effects and what to do if they experience side effects, and 25% more women said they were told about other methods of contraception. The results from household surveys at baseline and endline suggest that CHWs in this model made a significant contribution to family planning in the region.

## Abstract (precis)

Volunteer community health workers (CHWs) administered injectable contraceptives to women in the community for a small fee while providing counseling and referrals for other methods. Over nearly 3 years, more than 600 CHWs provided an estimated 15,410 injections. The model has the potential to improve sustainability of community-based distribution programs by incorporating social marketing principles to partially recover commodity costs and compensate CHWs.

## INTRODUCTION

Meeting the contraceptive needs of rural women in sub-Saharan Africa remains a challenge. Family planning services are predominantly offered through public health facilities, but access is limited by distance to facility, quality of services, availability and affordability of contraceptive methods, and medical or legal barriers.^1^^–^^4^ There is strong evidence to support the hypothesis that a country's contraceptive prevalence is directly associated with measures of access to both individual methods and a balanced method mix.^1^^,^^5^^–^^7^

In 2011, injectable contraceptives were the most commonly used method among married women in sub-Saharan Africa,^8^ and this trend has continued.^9^ Using multivariate models, Skiles et al. examined the effect of access to contraceptive services on injectable use and demand for family planning in Malawi and found that the probability of injectable use among rural women with the most access, as measured by distance alone and distance combined with supply reliability, was 7–8 percentage points higher than among rural women with the least access.^7^

Community-based distribution (CBD) programs that rely on community health workers (CHWs) have been successful in increasing access to and use of injectable contraceptives, particularly in settings where unmet need is high, access is low, and geographic or social barriers to use of services exist.^7^^,^^10^^–^^14^ A major strength of CBD programs is that they bring information, services, and supplies to women and men in the communities where they live and work. Integrating CHWs into health systems is considered a high-impact practice for family planning,^15^ yet funding and sustaining such programs remain a challenge given the cost of training and supervising CHWs who are often dispersed across geographic regions.^16^

Community-based distribution programs have been successful in increasing access to and use of injectable contraceptives, but funding and sustainability of such programs remain a challenge.

Ethiopia has made notable progress in increasing awareness and knowledge of family planning and is considered a success story among funders and program planners. Favorable political will, generous donor support, public-private partnerships, and the government's establishment of a Health Extension Worker (HEW) program have been identified as key factors in this success.^17^ The Government of Ethiopia launched the HEW program in 2004^18^ to deploy salaried health care providers to serve the primary health care needs of rural communities. The HEWs have a tenth grade education or more and receive training for 18 months. Two HEWs are based in each rural health post and provide some community outreach, such as vaccination campaigns.^19^^,^^20^

Starting in 2007, the government allowed HEWs to administer injectable contraceptives. This likely contributed to the doubling of injectable contraceptive use among women of reproductive age, from 6.8% in 2005 to 14.0% in 2011.^17^ In 2009, the government began training HEWs in the insertion of contraceptive implants. Subsequently, implant use increased from 0.2% in 2005 to 3.4% in 2011.^17^^,^^21^^,^^22^ Though HEWs have been an important addition to the public health sector, provision of family planning is just one of 16 basic health services they deliver to large, widespread populations, which may present limitations in family planning outreach efforts. Meanwhile, unmet need for family planning among rural women (28.6%) is almost double that of urban women (15.5%), with a wide gap in total fertility rate depending on urban (2.6) or rural (5.5) residence.^22^

In Ethiopia, unmet need for family planning among rural women is double that of urban women.

Consequently, volunteer CHWs can still be a valuable resource for bridging outreach activities and health post services. They can support HEWs delivering family planning information and services, serving as extensions of the health posts for remote areas, which is important given the high percentage of the Ethiopian population living in rural areas (81%).^22^ CHWs are also necessary given the multifaceted clinical roles HEWs have to assume due to health care worker shortages in the country. With declining fertility preferences and high unmet need for contraception, there is an opportunity to optimize CHWs and shift or share the task of family planning provision, including injectable contraceptives.

Recognizing a continued need for community-based access to the injectable contraceptive depot medroxyprogesterone acetate (DMPA), the University of California at Berkeley Bixby Center for Population, Health and Sustainability, in conjunction with Mekelle University College of Health Sciences, the Women's Association of Tigray, and the Tigray Regional Health Bureau, developed a service delivery model that combined CBD with social marketing. This model grew from a desire to scale up a pilot study where CHWs were successfully trained to provide injectable contraceptives in Tigray, Ethiopia.^12^ We incorporated private-sector strategies such as willingness-to-pay, social marketing, and a drug revolving fund, with the aim of creating a sustainable contraceptive service delivery model that used CHWs as rural social marketing agents to distribute DMPA. Long-term availability of DMPA, the supply and distribution of DMPA, and reduced CHW attrition were key factors in designing the model.

We tested a service delivery model that combined community-based distribution of injectable contraceptives with social marketing.

The purpose of this article is to illustrate the impact of the program model on reducing barriers to DMPA access in Tigray and increasing commodity security in rural communities. We draw on experiences from scaling up this model to describe lessons learned and factors that contributed to implementation of the program in Ethiopia, with hopes of informing family planning strategies and practical application in other settings.

## PROGRAM DESCRIPTION

### Program Model

The Figure depicts the program model implemented in this study in the Central and Southern rural zones of Tigray, Ethiopia, between September 2011 and June 2014. First, 2 CHWs were selected and trained from each participating *kebele* (village) to administer injectable contraceptives and provide counseling and referrals to the facility for other contraceptive methods. After the training, CHWs were provided with a supply of 25 DMPA injections in the form of a microloan from a drug revolving fund. The drug revolving fund credited CHWs 3 birr (US$0.17) for each dose of DMPA, which was the subsidized cost of 1 dose of DMPA purchased from DKT-Ethiopia, the social marketing agency. CHWs provided each DMPA injection to women for 5 birr (US$0.29). This amount was determined using willingness-to-pay data from the pilot study,^12^ which was confirmed as acceptable during baseline data collection.^23^ Of the 5 birr payment received for each injection administered, CHWs returned 3 birr to the drug revolving fund and kept the remaining 2 birr (US$0.12). CHWs also had discretion to provide injections for free to women who could not afford to pay and were not able to reach a facility for free injections; the CHWs were not held accountable for the cost of the drug in these instances. Funds returned to the system through sales of DMPA were managed by the Women's Association of Tigray and used to purchase additional DMPA from DKT-Ethiopia.

Of the 5 birr that CHWs charged women for each DMPA injection, the CHWs returned 3 birr to the drug revolving fund and kept the remaining 2 birr.

**FIGURE:**
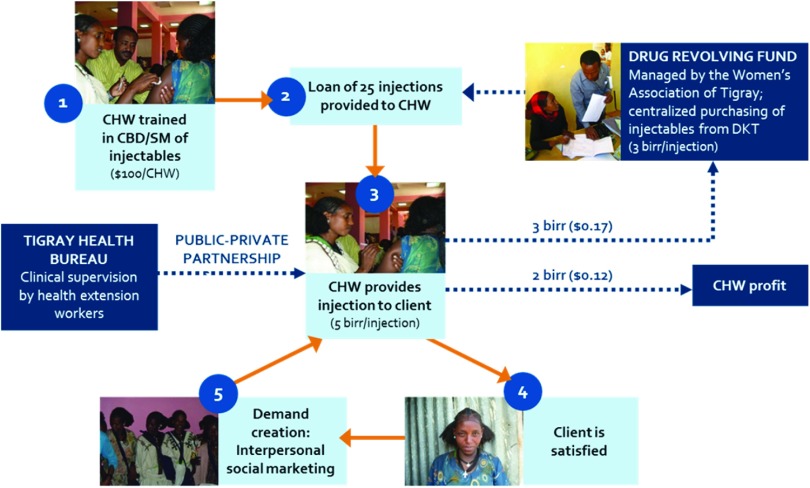
Program Model for Combining Community-Based Distribution and Social Marketing in Tigray, Ethiopia Abbreviations: CBD, community-based distribution; CHW, community health worker; SM, social marketing.

These doorstep services were intended to increase client satisfaction. Social marketing, an approach that integrates marketing concepts with other approaches such as subsidized products to influence behaviors that benefit individuals and communities for the greater social good, was used to create awareness of the product and generate demand for services (Box). In fact, CHWs were considered rural social marketers generating demand through both conventional social marketing techniques and methods tailored more to rural audiences. The injectable contraceptive product of DKT-Ethiopia used in this project is marketed under the brand name *Confidence*, which helped with visibility in the community. CHWs received a marketing poster for display in their homes, but their services were also promoted through door-to-door outreach, community meetings, and word-of-mouth among women. The rationale behind the program model and initial roll out is described in more detail by Prata et al.^25^

CHWs used social marketing techniques, tailored to rural audiences, to promote demand for family planning.

BOX **Social Marketing**

**Social marketing** seeks to develop and integrate **marketing** concepts with other approaches to influence behaviors that benefit individuals and communities for the greater **social** good, such as anti-smoking campaigns and use of contraception for better maternal and child health. Unlike commercial marketing in which the primary aim is to reap financial benefits, the focus of social marketing is on the social good.

 Social marketers must create competitive advantage by constantly adapting to and instigating change. Market changes are likely to be more successful if actions are guided by knowledge of the forces shaping market behaviors and insights that enable the development of sustainable competitive advantages.

 In the family planning field, it became apparent that social marketing of contraceptives since the 1980s has not only increased awareness, acceptability, and use of modern contraceptives in developing countries but also overcome logistic problems in service delivery.^24^ This strategy has the ability to reach large numbers of potential clients, and it markets contraceptives as common consumer products. Each contraceptive social marketing program is built around a theme tailored to meet specific cultural, social, and management requirements. The primary target populations are those who cannot afford regular commercial products and those who are not adequately reached by government programs.

### Selection and Training of CHWs

CHWs, formerly called community-based reproductive health agents (CBRHAs) in Ethiopia, are volunteer community lay health care workers who have been mobilized to provide basic family planning education, services, and referrals in rural areas. The Women's Association of Tigray selected the CHWs for this program (2 per *kebele*), who came from the existing pool of CBRHAs in the region. Nurses and clinical officers from Mekelle University facilitated the training sessions under the supervision of program staff from the Bixby Center and Mekelle University. Each training session lasted 4 days, including ethical, clinical, and logistical components. Participating CHWs first practiced their injection techniques on oranges and learned how to dispose of the used needles following infection prevention procedures. After much practice, CHWs administered distilled water injections to each other until they were able to demonstrate proficiency. CHWs were trained in screening procedures using a checklist intended for use by clinical and nonclinical health care providers, including community-based distributors, which was developed by FHI 360.^26^ They were also trained on procedures for reporting side effects and adverse events, as well as referring clients to facilities for other methods. Business components such as requesting payment, procurement of DMPA, and basic logistics to avoid stock-outs were also included in the training. CHWs were trained on how to counsel on all family planning methods but were trained to provide only DMPA. Upon completion of the training, CHWs were recognized by the Tigray Regional Health Bureau as qualified DMPA providers.

### Clinical Supervision

The Tigray Regional Health Bureau was engaged as a partner in monitoring and evaluation to ensure supervision efforts during the study period were an extension of the public health system rather than a parallel system. The ultimate goal was to promote compliance with family planning practice standards and assure delivery of high-quality services that continued after the completion of the study.

Thus, 2 levels of supportive supervision were employed. The first level of supervision, as well as the most important, was conducted by the HEWs. At the time of program implementation, HEWs were already meeting with existing *kebele* CHWs to discuss other community health initiatives. The HEWs provided the most obvious link between our program CHWs and the public health system. HEWs collected data on service statistics and discussed any clinical issues with CHWs including referrals for long-acting methods such as implants. These data were reported to the maternal and child health (MCH) experts at district health offices on a monthly basis. MCH experts reported monthly data to research study coordinators.

Health extension workers (HEWs) from the Tigray Regional Health Bureau supervised the CHWs.

As a second level of supervision, MCH experts and research study coordinators scheduled frequent visits to CHWs to provide both supervision and to learn about the experiences of participating CHWs. CHWs that were effective as well as those who were ineffective participants in the program were selected for these in-depth visits, as it was through such visits that research staff gained insights into enablers and barriers to success.

All levels of supervision were done once a month in person at minimum, followed in some cases by cell phone communication if needed.

### Drug Revolving Fund

A drug revolving fund was included in the program model to promote geographical equity of access to DMPA. Procurement of DMPA occurred at the regional level with distribution to 18 satellite locations in each participating *woreda* (district). The Women's Association of Tigray provided financial supervision of the CHWs. As a civil society organization with a presence in each *woreda* and experience overseeing microloans, the Women's Association of Tigray was in a position to provide this supervision for the foreseeable future and ensure the timely resupply of commodities to the CHWs. At the same time, with a central office, the organization was able to manage the drug revolving fund and procure DMPA at a lower rate by purchasing on behalf of the region. DMPA was distributed to the local office located in each *woreda* center. A leader from the Women's Association of Tigray collected money from and reviewed the financial records of CHWs, replenished CHWs' stock, and managed *woreda*-level drug revolving fund supply and financial records. These leaders established bank accounts for deposits, and transfers were made to the regional office prior to procurement of additional DMPA.

### Social Marketing in a Rural Setting

Social marketing is generally limited to urban settings where exposure to the mass media is high, private shops and pharmacies are common, and demand for contraception is strong.^25^^,^^27^ However, recognizing that the foundation for social marketing existed in the rural setting, the program model incorporated social marketing into standard CBD. For one, the private sector is increasingly meeting the needs of poor populations. At the same time, existing community networks could be tapped through CHWs while retention could be improved with compensation from sales. Consequently, social marketing tactics were adapted for the rural context. For example, while the product was branded and CHWs were given marketing posters as is typical with social marketing, the CHWs also promoted their services through community meetings and door-to-door marketing.

## METHODS

### Study Design and Sampling

We used a pre- and post-intervention study design. A multistage, cluster random sampling design was used to conduct a representative survey of women of reproductive age (15–49) at baseline (September 2011) and endline (May 2014) in the Central Zone of Tigray, Ethiopia. The total final samples consisted of 1,490 women for the baseline survey^23^ (99% response rate) and 1,501 women for the endline survey^28^ (100% response rate). Participating CHWs were also surveyed at baseline and endline, with a 99% response rate (N=621) at baseline and a 74% response rate (N=466) at endline. The endline response rate reflects those CHWs who participated at a large program review meeting where the survey was implemented. Thus, 26% is not a true refusal rate but rather reflects the number of CHWs absent from the meeting. Additionally, 87% of trained CHWs (N=545) submitted programmatic DMPA provision data at endline.

### Project Setting

Tigray is made up of 5 rural zones divided into 47 *woredas*. Each *woreda* is further divided into approximately 20–25 *kebeles*. Each *kebele* has a population size of approximately 5,000 people or 1,000 households. Two zones participated in the project: Central and Southern. The Central and Southern Zones are comprised of 10 and 8 *woredas*, respectively, with an estimated 239,626 and 197,215 women of reproductive age in each zone, respectively, based on projected population growth since the 2007 census.^29^

### Incremental Implementation

The project was implemented between September 2011 and June 2014, with a total of 626 CHWs recruited and trained from 18 *woredas*. During the first 12 months, the project was implemented in just 3 districts with 139 CHWs to test the service delivery model and address any challenges before scaling up sixfold.^25^ Starting in the second year of the project, scale-up was accelerated with 4 trainings of 100–150 CHWs each. The intervention was incrementally scaled up to all *woredas* in the Central and Southern Zones between October 2011 and October 2013.

### Data Collection and Analysis

Programmatic data on the number of clients, number of injections provided, and money collected were gathered from CHWs and supervisors, aiding the program team in determining the feasibility, cost, and sustainability of scaling up the intervention. A provider characteristics survey was completed by CHWs at the time of training and after implementation of the program to gather demographic data and capture information on their experience with the program.

Comparisons between the baseline and endline household survey data were used to measure the impact of the intervention. Student's *t* tests for comparison of 2 proportions were estimated for indicators comparing baseline and endline. Significance was established at *P* value of .05. The surveys captured demographic, fertility, and contraceptive use patterns among the target population to assess changes in family planning knowledge, access, use, and preferences. The surveys also included questions regarding knowledge, attitudes, and practices related to DMPA. Key indicators from baseline and endline reports were compared to determine changes over the course of the intervention. Human subjects approval was provided by the Center for Protection of Human Subjects (CPHS) at the University of California Berkeley (CPHS Protocol IDs 2011-07-3465 and 2014-02-5995).

All paper questionnaires from the provider characteristics surveys, as well as baseline and endline household surveys, were processed at the Bixby Center and entered into a database using Epi Info Version 3.5.3. A master file of programmatic data collected from CHWs at endline meetings was created by research staff. Research staff were able to verify data accuracy by reviewing the actual records of the CHWs, whereas the ongoing monthly data collection had limitations and concerns regarding accuracy and completeness. Additionally, due to differences in the Ethiopian and Gregorian calendar, programmatic data could not be accurately analyzed per month. All data analyses were conducted using Stata version 13.

## RESULTS

### Background Characteristics and Experience of CHWs

The CHWs (N=621) who completed the baseline questionnaire were, on average, 27.1 years old, and 60% were married or cohabiting. Almost all (98%) participating CHWs were women, and 67% identified farmer or housewife as their current occupation. Forty-two percent of participating CHWs had 5–9 years of education, while 41% completed secondary school or higher. The higher levels of education observed among program CHWs compared with the general population was a result of targeted recruitment of CHWs based on the characteristics we identified among successful CHWs in the first year of implementation, which included greater education. At the time of training, 26% of CHWs were currently providing family planning, with 5% stating they had ever provided a DMPA injection. Nearly all (93%) felt that providing DMPA injections would improve their services to the community; meanwhile, 96% and 87% of CHWs felt comfortable providing services to adolescents and unmarried women, respectively.

CHWs completed another survey at endline in June 2014 (N=466). Nearly 85% reported a leadership role in their community. While the CHWs largely identified having a leadership position in the Women's Development Army (61%), they also had positions in the Women's Association of Tigray (25%) and *kebele* government (7%); these categories were not mutually exclusive. On average, CHWs were 50 minutes walking distance from the nearest government health post and spent approximately 5 hours per week marketing DMPA. Many CHWs confirmed they had provided an injection to unmarried (58%) or adolescent women (63%). Most (75%) felt comfortable collecting payment for services. Meanwhile, most CHWs felt supported by project personnel (89%), HEWs (88%), and the Women's Association of Tigray (82%). Nearly all CHWs (95%) felt the community accepted the project, and 89% of married/cohabiting CHWs felt supported by their husband or partner.

Many CHWs confirmed they had provided a DMPA injection to unmarried or adolescent women.

### Programmatic Results

Between May 2014 and June 2014, 87% of trained CHWs (N=545) participated in endline data collection meetings and submitted final programmatic data. The number of months of data collected corresponded with the gradual training approach used over the course of project implementation. Consequently, while CHWs trained from the first 3 *woredas* had 30 months of programmatic data, CHWs in the last training had only 8 months of programmatic data. On average, 16 months of programmatic data were collected from participating CHWs.

Between October 2011 and June 2014, the CHWs served a total of 8,604 women and administered an estimated 15,410 DMPA injections. This is equivalent to providing 3,853 couple-years of protection (CYP). A substantial percentage (19%) of CHW clients were new to family planning and 25% were new to DMPA specifically. The majority (87%) of the DMPA injections were paid for at the time of provision. The costs of delivering contraceptive services were collected in 3 *woredas* where the project was first implemented. A cost analysis conducted with these data found the programmatic cost per CYP to be US$17.91, which included direct, indirect, and operating costs. The direct cost per CYP was US$2.96.^30^

Between October 2011 and June 2014, the CHWs administered an estimated 15,410 DMPA injections, equivalent to 3,853 couple-years of protection.

### Changes Over Time at the Community Level

The baseline and endline survey captured demographic, fertility, and contraceptive use patterns among the target population. Table 1 presents the background characteristics of all women ages 15–49 who participated in the surveys. None of the characteristics presented differed with statistical significance. At both baseline and endline, the majority of the sample population was under the age of 30, most women were married or cohabiting, and approximately half of each sample had received no formal education. The average number of living children among women in the sample population was 3.6 at baseline and 3.5 at endline, and desired number of children was 4.1 and 4.5 at baseline and endline, respectively. Most survey participants responded that they did want a/another child at baseline (60%) and endline (66%), but the desire for more children gradually declined as the number of living children reported by the participants increased (data not shown).

**TABLE 1.** Background Characteristics Among All Women of Reproductive Age at Project Baseline (September 2011) and Endline (May 2014), Tigray, Ethiopia

	Baseline (N=1490)	Endline (N=1501)
Age		
15–19	19.3	18.5
20–24	17.1	20.7
25–29	17.9	17.2
30–34	15.7	14.5
35–39	13.4	13.3
40–44	8.3	7.9
45–49	7.5	7.9
Marital status		
Never married	13.6	12.5
Married/cohabiting	72.3	76.4
Divorced/widowed	13.9	10.9
Education		
No education	53.6	48.4
1–4 years	13.2	14.3
5–9 years	22.4	25.9
Secondary or greater	10.6	11.1
Number of children ever born, mean	3.9	4.0
Number of living children, mean	3.6	3.5
Desired number of children, mean	4.1	4.5

Data reported as percentages unless otherwise specified.

Comparisons between baseline and endline showed substantial changes in contraceptive knowledge and prevalence, some of which is likely attributable to the program CHWs' activities. For example, between October 2013 and June 2014, women's knowledge of modern methods increased significantly (*P*<.005) for all methods except the rhythm method (Table 2). In addition, there was a 25% increase (*P*<.001) in contraceptive use among surveyed women, from 30.1% at baseline to 37.7% at endline, with DMPA use largely responsible for this increase (*P*<.001) (Table 3). The largest increase in DMPA use with statistical significance was found among women aged 15–24 (*P*<.001). Also, among all women of reproductive age, 8.3% preferred to receive contraception from a CHW at a baseline, whereas 31.1% (*P*<.001) preferred to receive contraception from a CHW at endline (Table 3).

**TABLE 2.** Changes in Knowledge of Contraceptive Methods Among Women of Reproductive From Project Baseline (September 2011) to Endline (May 2014), Tigray, Ethiopia

	Baseline (N=1490) %	Endline (N=1501) %	% Change	*P* Value
Female sterilization	21.0	33.8	61	<.001
Male sterilization	7.9	15.4	95	<.001
Pill	91.7	96.2	5	<.001
IUD	23.9	50.6	112	<.001
DMPA/injectables	96.1	97.9	2	<.01
Implants	69.8	88.6	27	<.001
Male condom	57.1	78.5	37	<.001
Female condom	16.5	26.3	59	<.001
LAM	30.1	34.2	14	<.05
Rhythm method	31.3	34.0	9	NS
Withdrawal	12.6	20.1	60	<.001
Emergency contraception	11.2	15.1	35	<.01

Abbreviations: DMPA, depot medroxyprogesterone acetate; IUD, intrauterine device; LAM, lactational amenorrhea method; NS, not significant.

**TABLE 3.** Changes in Key Family Planning Indicators Among Women of Reproductive Age From Project Baseline (September 2011) to Endline (May 2014), Tigray, Ethiopia

	Baseline No. (%)	Endline No. (%)	% Change	*P* Value
Unmet need	1077 (16.4)	179 (11.9)	–28	<.01
Currently using contraception	448 (30.1)	566 (37.7)	25	<.001
Currently using DMPA	307 (20.6)	408 (27.2)	32	<.001
By age group				
15–19	21 (7.3^a^)	39 (14.1)	93	<.01
20–24	53 (21.3)	99 (31.8)	49	<.01
25–29	91 (34.1)	93 (36.1)	6	NS
30–34	61 (26.1)	77 (35.5)	36	<.05
35–39	48 (24.0)	59 (29.5)	23	NS
40–44	21 (17.1^a^)	27 (22.7)	33	NS
40–49	7 (6.3^a^)	14 (11.9^a^)	88	NS
CHW as preferred source of contraception	124 (8.3)	467 (31.1)	275	<.001

Abbreviations: CHW, community health worker; DMPA, depot medroxyprogesterone acetate; NS, not significant.

a Estimate was based on less than 25 cases.

Contraceptive use among surveyed women had increased significantly by 25% between baseline and endline, from about 30% to 38%.

At endline, one-quarter of women using DMPA indicated that they received DMPA from CHWs (*P*<.001) (Table 4). The percentages were even higher among younger women, with 35% of women aged 15–24 and 46% of women aged 25–34 stating that CHWs provided their most recent DMPA injection (data not shown). In addition, there was a substantial change in women who preferred to receive DMPA from CHWs between baseline and endline, from 2.7% to 34.1% (*P*<.001) (Table 4). Finally, changes in quality of family planning markers from baseline suggest services had improved: nearly 50% (*P*<.001) more women reported being told about side effects (from 46.8% to 68.7%) and what to do if they experience side effects (from 43.5% to 63.1%), and over 25% (*P*<.001) more women reported being told about other methods of contraception (from 65.4% to 82.9%) (Table 5).

**TABLE 4.** Changes in Most Recent Source of DMPA Among Women Who Have Ever Used DMPA From Project Baseline (September 2011) to Endline (May 2014), Tigray, Ethiopia

	Baseline (N=662) %	Endline (N=840) %	% Change	*P* Value
Most recent source of DMPA				
Government hospital	1.1^a^	0.5^a^	−56.4	.19
Government health center	59.8	37.9	−36.7	<.001
Government health post	37.8	30.5	−19.4	<.001
CBRHA	0.8^a^	25.5	3085.0	<.001
Other	0.6^a^	0.0^a^	NA	NA
Preferred source of DMPA				
Government hospital	1.8^a^	1.4^a^	−20.6	.54
Government health center	51.4	41.4	−19.4	<.001
Government health post	39.9	53.8	34.9	<.001
CBRHA	2.7^a^	34.1	1161.1	<.001
Other	9.7	0.1^a^	−98.8	<.001

Abbreviations: CBRHA, community-based reproductive health agent; DMPA, depot medroxyprogesterone acetate.

a Estimate was based on less than 25 cases.

**TABLE 5.** Changes in Quality of Family Planning Service Markers From Project Baseline (September 2011) to Endline (May 2014) as Reported by Women of Reproductive Age Who Are Currently Using Contraception, Tigray, Ethiopia

	Baseline (N=448) %	Endline (N=566) %	% Change	*P* Value
Told about side effects	46.8	68.7	46.9	<.001
Told what to do if they experience side effects	43.5	63.1	45.0	<.001
Told about other methods	65.4	82.9	26.7	<.001

## DISCUSSION

Evidence suggests that CHW provision of contraceptives help to reduce barriers to family planning access due to CHWs' placement within the community. The important role CHWs play in changing norms and influencing traditional structures in rural Africa as respected community members should also not be overlooked when exploring the importance of contraceptive CBD programs.^31^^,^^32^ CBD has been shown to effectively meet the growing demand for contraceptives but still remains one of the more expensive modes of service delivery in sub-Saharan Africa.^33^ Additionally, lack of appropriate remuneration as balanced with responsibilities has implications for both motivation and the quality of work among CHWs.^34^ Consequently, political will and financing, including incentives to CHWs, must be addressed to ensure sustainability and scalability of CBD programs.^16^^,^^32^^,^^35^

This study demonstrated a model for incorporating CBD with social marketing that offers an opportunity for expanding rural community access to DMPA injectables while recovering some program costs and compensating volunteer CHWs with proceeds from contraceptive sales. In addition, the model has the potential to increase quality of services by providing more women with information on a wide range of contraceptives, side effects, and what to do if they experience side effects. The willingness and ability of CHWs in this study to serve adolescents was a particularly important finding, given that unmarried and younger women in sub-Saharan Africa often face barriers to receiving family planning services, including barriers related to provider discrimination and unnecessary medical restrictions based on, for example, age or marital status.^36^^,^^37^

Incorporating community-based distribution with social marketing offers an opportunity to expand rural access to DMPA injectables while recovering some program costs and compensating volunteer CHWs.

However, anecdotal evidence from CHWs collected during supportive supervision visits indicated that adolescent clients were less likely to pay for services than older clients, which is not surprising given that youth might have less access to cash in rural Tigray. The drug revolving fund was included in the program to enable services for all women, even those without means to pay or reach a health facility for free services. For the most part, CHWs referred women to health posts for services if they were unable to pay, but CHWs were given the discretion to provide free DMPA injections without them having to reimburse the drug revolving fund the cost of the injection. This drug revolving fund was set up to ensure continued DMPA stock in the community, but its long-term sustainability is dependent on women's response to paying for DMPA and the number of payment exemptions made.

While the majority (87%) of injections were paid for over the 3-year implementation period, the continued desire and ability of women to seek out and pay for family planning is affected by socioeconomic factors. The women marketed through this intervention are predominantly rural farmers and therefore at risk for unpredictable economic shocks, leaving families to prioritize food and the health of existing children, rather than family planning commodities. Thus, the health and future outlook of the drug revolving fund is theoretically in danger if women are not able to pay the current price of the injection. The drug revolving fund would also be threatened if the price of a DMPA injection increased to an amount that it is no longer affordable to women. As noted, DKT-Ethiopia subsidized the DMPA product for the project (US$0.17 per unit); however, a 2012 analysis from the United Nations Population Fund (UNFPA) found the average cost of 1 injection globally was $0.86.^38^ Thus, it is important to consider this price differential when thinking about the findings from this study.

The total number of injections (15,410) provided over the course of the 3-year period was not as high as might be expected with more than 600 CHWs participating in the project. This is partially due to the protracted scale-up of the model over a 2-year period, but it also indicative of the challenges associated with family planning service provision in rural areas. In addition to geographic isolation of small communities, the decision to adopt family planning and people's desired family size also influence demand for services. However, the growing preference among women to receive contraception from CHWs between baseline and endline does suggest the continued potential of this model and the importance of providing community-based access to family planning.

Data from the CHWs indicated that many clients who received their first and second injections from the HEWs received their third and fourth injections from CHWs. No unintended pregnancies from missing injections were reported by CHWs during the study period. A few CHWs did report that after receiving initial DMPA injections for a fee in the community from the CHWs, some women subsequently went to health posts for free reinjections. Unfortunately, we do not have data from health posts to verify if this was actually the case. However, a forthcoming and more detailed analysis of population-based data on continuation rates seem to indicate that women report getting the injections. Similarly, CHWs reported making many referrals to health posts for other contraceptive methods, largely implants; however, we cannot verify the referrals and subsequent method adoption with health post data.

While robust recruitment and training strategies increased engagement, commitment, and comprehension among CHWs, compensation remains an important factor to understand. The number of injections provided per CHW directly impacts her compensation, which was a factor that influenced the program design. CHWs received 2 birr ($0.17) per paid injection, but there was not substantial evidence to determine the compensation that would motivate CHWs to continue providing DMPA beyond the project. There is actually a strong legacy of volunteerism for community health in Ethiopia,^39^ and particularly in Tigray, which may have contributed to the low attrition of CHWs, even given small profit margins. Nonetheless, the importance of incentivizing CHWs to improve retention and performance has been well documented^16^ and should be further explored with regards to this contraceptive service delivery model in Tigray.

It is also critical to understand this service delivery model within the larger context of family planning service delivery in Tigray. For example, with a social marketing model, it is also important to determine the material contribution beyond injections, including increases in knowledge and change in community norms surrounding family planning. In the 2011 Ethiopia Demographic Health Survey, 23% of women and men of reproductive age in Tigray had never heard or seen a family planning message. Moreover, 64% of women of reproductive age who were not using contraception reported that they had never discussed family planning with a field worker or at a health facility.^22^ By training health workers in the community to provide messages on the benefits of family planning, community beliefs are influenced and demand for family planning will likely grow over time as myths are dispelled and side effects understood.

Another underlying key to the success of this model was that it expanded upon the existing health service delivery platform in Tigray, essentially creating a public-private partnership between the health posts and CHWs. Linkages with the community were supported through this model by including the Women's Association of Tigray to both manage the drug revolving fund and provide financial supervision of the CHWs. The organization has a strong presence from the regional level down to each *kebele* in Tigray. This allowed for both centralized purchasing of DMPA, as well as localized management of microloans to CHWs. The model also improved community linkages with public health services through referrals and monitoring. HEWs supervised CHWs' clinical activities, which enabled project monitoring with minimal additional impact on the health system. Most CHWs were leaders in the Women's Development Army and therefore already met with HEWs on regular basis to share updates on their activities.

Expanding on the existing health service delivery platform likely contributed to the success of the public-private partnership model.

Finally, the program model expanded services of the public health system for a fee to women who were willing to pay for the convenience and privacy the CHWs provided. With monitoring systems already incorporated into the activities of the Tigray Regional Health Bureau, the program was able to continue after the 3-year funding period ended with minimum transition efforts.

### Limitations

The Ethiopian calendar is substantially different than the Gregorian calendar. CHWs recorded data following the Ethiopian calendar, with the intention that the monitoring and evaluation program team would translate these data into a 12-month calendar. The transferring of data from CHWs to program staff was prone to error because of calendar issues combined with untimely submission of data due to remote location of many CHWs. Ultimately, we limited our results to total injections, which did not permit us to analyze trends and month-by-month breakdown between *kebeles* and CHWs—key information that could have helped inform future programming.

There were also resupply and data collection challenges related to the drug revolving fund. The Women's Association of Tigray was selected to manage the drug revolving fund given its experience in supporting programs that involved microloans at the community level. However, given the time demands and complexity of implementing a regional drug revolving fund with satellites in 18 *woredas*, the organization required more experience and logistical support than provided and the records on resupply and repayment were challenging to triangulate with records from CHWs. At the same time, with a nascent drug revolving fund, it is difficult at this time to draw real conclusions about its long-term sustainability and whether the CHWs have sufficient revenues to replenish the DMPA stocks. Consequently, further research on the drug revolving fund is necessary. Nonetheless, the results from household surveys at baseline and endline suggest that CHWs in this model made a significant contribution to family planning services in the region.

## CONCLUSION

By addressing women's willingness-to-pay for contraception while incorporating social marketing and a drug revolving fund, this contraceptive service delivery model has potential to increase sustainability by training volunteer CHWs to become rural social marketing agents of DMPA injectables. This model can help accelerate contraceptive adoption, especially among rural women in sub-Saharan Africa. However, long-term sustainability of CBD should be addressed when designing programs, particularly the key challenges of managing attrition among CHWs and maintaining supply of contraceptives.

## Notes

**Peer Reviewed**

**First Published Online:** 2017 Mar 8
